# Pharmacokinetic characteristics of golidocitinib, a highly selective JAK1 inhibitor, in healthy adult participants

**DOI:** 10.3389/fimmu.2023.1127935

**Published:** 2023-04-03

**Authors:** Kan Chen, Xiaoduo Guan, Zhenfan Yang, Yue Zhou, Ziyi Liu, Xueyuan Deng, Donghong Liu, Pei Hu, Rui Chen

**Affiliations:** ^1^ Dizal Pharmaceuticals, Shanghai, China; ^2^ Clinical Pharmacology Research Center, Peking Union Medical College Hospital, State Key Laboratory of Complex Severe and Rare Diseases, NMPA Key Laboratory for Clinical Research and Evaluation of Drug, Beijing, China; ^3^ Beijing Key Laboratory of Clinical PK & PD Investigation for Innovative Drugs, Chinese Academy of Medical Sciences & Peking Union Medical College, Beijing, China

**Keywords:** golidocitinib, JAK1 inhibitor, pharmacokinetics, ethnicity, food effect

## Abstract

**Background:**

Golidocitinib is an orally available, potent and highly selective JAK (Janus kinase)-1 inhibitor of JAK/STAT3 signaling under clinical development for the treatment of cancer and autoimmune diseases. The objectives of the two reported studies were to investigate the pharmacokinetics (PK), safety, and tolerability of golidocitinib in healthy Chinese participants as compared to those healthy Western participants, as well as the food effect exploration.

**Methods:**

Two phase I studies (JACKPOT2 and JACKPOT3) were conducted in USA and China, respectively. In JACKPOT2 study, participants were randomized into placebo or golidocitinib arm in single-ascending dose cohorts (5 - 150 mg) and multiple-ascending dose cohorts (25 - 100 mg, once daily) for 14 days. In the food effect cohort, golidocitinib (50 mg) was administrated shortly after a high-fat meal (fed conditions) as compared to under fasting conditions. In JACKPOT3 study conducted in China, participants were randomized to placebo or golidocitinib arm in single-ascending dose cohorts (25 - 150 mg).

**Results:**

Exposure of golidocitinib generally increased in a dose-proportional manner across a dose range of 5 mg to 150 mg (single dose) and 25 mg to 100 mg (once daily). High-fat food did not alter the PK of golidocitinib with statistical significance. Low plasma clearance and extensive volume of distribution characterizes PK of golidoctinib, and long half-life across the dose levels supported once daily dosing. The inter-ethnic difference in primary PK parameters was evaluated. The result suggested slightly higher peak plasma concentrations (C_max_) but comparable area under the plasma concentration-time curve (AUC) was observed in Asian (Chinese) subjects as compared to Caucasian and/or Black subjects, while it was not considered clinically relevant. Golidocitinib was well tolerated without Common Terminology Criteria for Adverse Events (CTCAE) grade 3 or higher drug-related treatment emergent adverse events (TEAE) reported.

**Conclusion:**

No noticeable inter-ethnic difference was observed among Asian, Black, and Caucasian healthy subjects in anticipation of the favorable PK properties of golidocitinib. The effect of food on the bioavailability of golidocitinib was minor following a single oral administration of 50 mg. These data guided to use the same dose and regimen for multinational clinical development.

**Clinical trial registrations:**

https://clinicaltrials.gov/ct2/show/NCT03728023?term=NCT03728023&draw=2&rank=1, identifier (NCT03728023); http://www.chinadrugtrials.org.cn/clinicaltrials.searchlistdetail.dhtml, identifier (CTR20191011).

## 1 Introduction

The Janus kinases (JAKs) are a family of intracellular tyrosine kinases of the class I and II receptor superfamily that consists of four members: JAK1, JAK2, JAK3, and Tyrosine kinase 2 (TYK2). Activation of JAKs occurs when a cytokine binds to its receptor, inducing multimerization of receptor subunits that brings JAKs into proximity with each other for transactivation. Activated JAKs then phosphorylate signal transducer and activation of transcription (STAT) proteins, resulting in their dimerization. Phosphorylated STAT dimers translocate to the nucleus to modulate the expression of the JAK/STAT target gene. Thus, JAKs play a critical role in the JAK/STAT signaling cascade to transmit information from extracellular cytokine cues to the nucleus, leading to DNA transcription and expression of genes involved in immunity, proliferation, differentiation, apoptosis, and oncogenesis (1).

The discovery of numerous cytokines underlying the pathogenesis of allergic, inflammatory, and autoimmune disorders has provided the basis for developing drugs targeting cytokines and cytokine receptors for treating autoimmune disorders. Many of these cytokines, such as Type I and Type II cytokines, signaling *via* JAK/STAT pathway is well established in various genetic models from mutagenized cell lines engineered with human genes and knockout mice (2). A critical role of JAK/STAT in Type I and Type II cytokine signalings, such as gamma chain (γc) cytokines (IL-2, IL-4, IL-7, IL-9, IL-15, and IL-21), IL-6, IL-12, IL-23, and IFNγ, strongly argue that interfering with the activity of these kinases could lead to a new class of immunomodulatory (2, 3). Several JAK inhibitors have been approved for the treatment of rheumatoid arthritis (RA) and ulcerative colitis (UC), and several JAK inhibitors are at various stages of clinical development for the treatment of autoimmune diseases (4). These pieces of evidence support the importance of the JAK/STAT pathway in autoimmune diseases. Constitutive activation of JAK/STAT pathways is also associated with a wide variety of malignancies and are implicated in tumor escape from chemo- and targeted- therapies (5, 6). Inhibition of the JAK/STAT pathway by ruxolitinib, a JAK1/JAK2 inhibitor, showed promising anti-tumor activity in patients with advanced T cell malignancies, especially tumors with JAK/STAT pathway aberrations (7).

Moreover, less optimal efficacy is likely caused by the short half-life of ruxolitinib and less durable modulation of JAK/STAT. Amongst the JAK family kinases, JAK1 has been shown to be the primary driver of STAT3 phosphorylation and activation of signaling pathways. Thus, targeting JAK/STAT3 pathway is an attractive therapeutic approach for patients with Peripheral T-cell lymphoma (PTCL). Non-clinical efficacy and PK/PD studies of golidocitinib (also named as AZD4205 or DZD4205) in xenograft model allow for increased and longer suppression of JAK1/STAT3 signaling and subsequent better anti-tumor activity as compared to ruxolitinib (Jason G. 7, 8).

Golidocitinib is an investigational, potent, ATP-competitive, and JAK1 selective inhibitor. It exhibits greater than 200 folds of selectivity to JAK1 over other JAK family kinases and has a favorable selectivity profile across the kinome (9). Golidocitinib demonstrated *in vitro* activities against cytokine-induced pSTATs in human blood cells as well as exhibiting promising *in vivo* efficacy in autoimmune disease and tumor xenograft models in animals.

Golidocitinib has been assessed in two phase I clinical studies, JACKPOT2 (NCT03728023) and JACKPOT3 (CTR20191011). These two studies were conducted in the USA and China, respectively, to compare the PK, safety, and tolerability of golidocitinib among major ethnic groups (Caucasian, Black, and Asian). Golidocitinib is currently being evaluated in a phase I/II study in patients with refractory or relapsed T cell lymphoma (r/r PTCL) (JACKPOT8, NCT04105010) and cutaneous T cell lymphoma (CTCL) (JACKPOT15, CTR20212475) as well as potentially autoimmune diseases. The PK information from these two clinical trials pharmacokinetically lays the foundation to support its global development strategy.

## 2 Methods

### 2.1 Phase I study in the USA (JACKPOT2, NCT03728023)

#### 2.1.1 Study design

**Figure 1:**
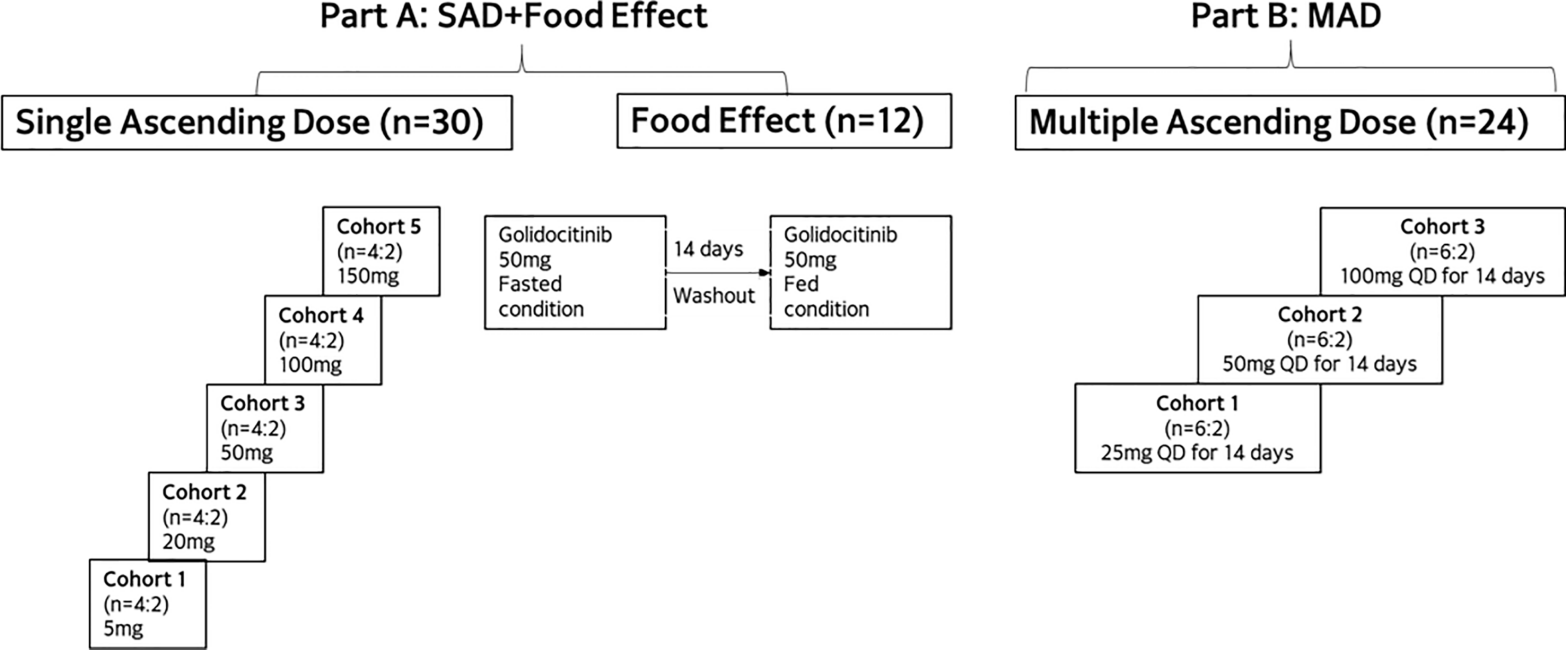
Study design of JACKPOT2.

This was a Phase I, randomized, double-blinded, placebo-controlled study to assess the safety, tolerability, and PK of golidocitinib in healthy participants in the USA. This study included two parts: part A (single-ascending-dose [SAD] and food effect [FE] cohorts) and part B (multiple-ascending-dose [MAD] cohorts). Please refer to **Figure 1** for the study flowchart.

A total of 42 and 24 healthy participants were enrolled in part A and part B, respectively. In part A, a total of 30 participants were randomized into 5 SAD cohorts (n = 6 per cohort) and received a single dose of golidocitinib at different dose levels or a matching placebo in a 4:2 ratio. In the food effect cohort, a total of 12 participants were enrolled and received the study drug under fasted or fed conditions, with a sequential design. The 50 mg dose was selected based on available safety data after the completion of the SAD cohorts, and also on the outcomes from PK/PD modeling for potential efficacious dose for autoimmune disease. On Day 1, participants took a single dose of golidocitinib under fast condition. After a 14-day wash-out period, on Day 15, participants received another single dose of golidocitinib immediately after a standard high-fat meal. Food recipes and contents are consistent with that described in FDA Guidance for Food-Effect Bioavailability studies (10). For the fed condition, keeping fasted for at least 10 hours, participants started ingestion of high-fat meal 30 minutes prior to administration of golidocitinib. They had to finish the meal within 30 minutes or less, and no food was allowed for at least 4 hours post-dose. In part B, a total of 24 participants were randomized into 3 MAD cohorts, with 8 in each cohort (active:placebo = 6:2). In these cohorts, participants received golidocitinib at different dose levels or placebo once daily dosing for 14 days. Dose escalation was based on available PK and safety data in the previous cohorts.

#### 2.1.2 Participants

Healthy male and female Black or Caucasian participants were aged 18~45 years old with a body mass index (BMI) between 18 and 30 kg/m^2^ (inclusive). Participants were not included in the study if they had a clinically significant history of hematological, renal, lipid, endocrine, pulmonary, gastrointestinal, cardiovascular, hepatic, psychiatric, neurologic, or allergic diseases; history of infections; received a live vaccine within three months before the first dose of golidocitinib; no over-the-counter or prescription medications within 14 days or five half-lives (whichever is longer), prior to receiving golidocitinib. Nicotine use > 1/2 pack per day of cigarettes or equivalent was prohibited 30 days prior to receiving golidocitinib and through the duration of the study. Alcohol was prohibited from 72 hours before admission for any study-related procedures and during the residential period. Participants fasted from at least 10 hours before the planned starting dose and remained fasted for 4 hours post-dose (except water for thirst).

#### 2.1.3 PK sampling and bioanalytical assays

In the SAD cohorts, serial blood samples were obtained on Day 1 at pre-dose, and at 0.5, 1, 1.5, 2, 3, 4, 6, 10, 14, 24 (Day 2), 48 (Day 3), 72 (Day 4), 120 (Day 6), 168 (Day 8) and 216 (Day 10) hours post-dose for plasma PK analysis. A similar sampling schedule was undertaken for the MAD cohorts on Day 1 and Day 14, with additional trough samples obtained on Days 5, Day 8, and Day 11. In the MAD cohorts, urine samples were collected on Day 14 during the collection intervals of 0-4, 4-10, and 10-24 hours following the last dose for urine PK analysis. Plasma and urine samples were analyzed by Frontage Laboratories Inc. (New Jersey, USA) using a validated LC/MS/MS bioanalytical method to determine golidocitinib concentrations. Cross validation in plasma was successful between Frontage Laboratories Inc. (New Jersey, USA) and Frontage Laboratories Inc. (Shanghai) using clinical samples and spiked quality control samples.

#### 2.1.4 Safety assessments

Safety assessments included monitoring of adverse events/serious adverse events, clinical safety laboratory tests (clinical chemistry, hematology, coagulation, and urinalysis tests), 12-lead ECG, vital signs (blood pressure, pulse rate, and respiratory rate), and pulmonary function. All adverse events were coded using the Medical Dictionary for Regulatory Activities (MedDRA), version 22.0. The CTCAE 5.0 was used to define the intensity of AEs.

#### 2.1.5 Pharmacokinetic assessments

Non-compartmental PK analysis for golidocitinib concentrations was performed using Phoenix WinNonlin (Certara USA Inc., version 8.1).

To test dose-proportionality, log-transformed C_max_, AUC_0-t_, and AUC_0-inf_ and log-transformed dose were analyzed using a power model: Ln (PK parameter) = α + β * Ln (dose). α and β were the estimated intercept and slope parameters, respectively; The 90% CIs around the slopes (β) from each of these regression analyses were obtained from the model and presented, and plots of the log-PK parameter by log-dose were displayed. Dose proportionality was concluded if the 90% confidence interval for the ratio of dose-normalized, geometric mean values (R_dnm_) was contained completely within the range of 0.8 - 1.25 (11, 12).

For food effect assessment, log-transformed PK parameters (AUC_0-t_, AUC_0-inf_, and C_max_) of golidocitinib were analyzed using a mixed-effect model including terms for study treatment (fasted and fed) as fixed effects, and participants nested within treatment as a random effect. The CIs on the ratios of untransformed PK parameters were derived through a reverse transformation of the 90% CIs for the differences of the means in the log scale from the mixed-effect model. Least-squares geometric means, a ratio of the geometric means and 90% CIs on the ratio of fed to fasted were presented.

### 2.2 Phase I study in China (JACKPOT3, CTR20191011)

#### 2.2.1 Study design

**Figure 2:**
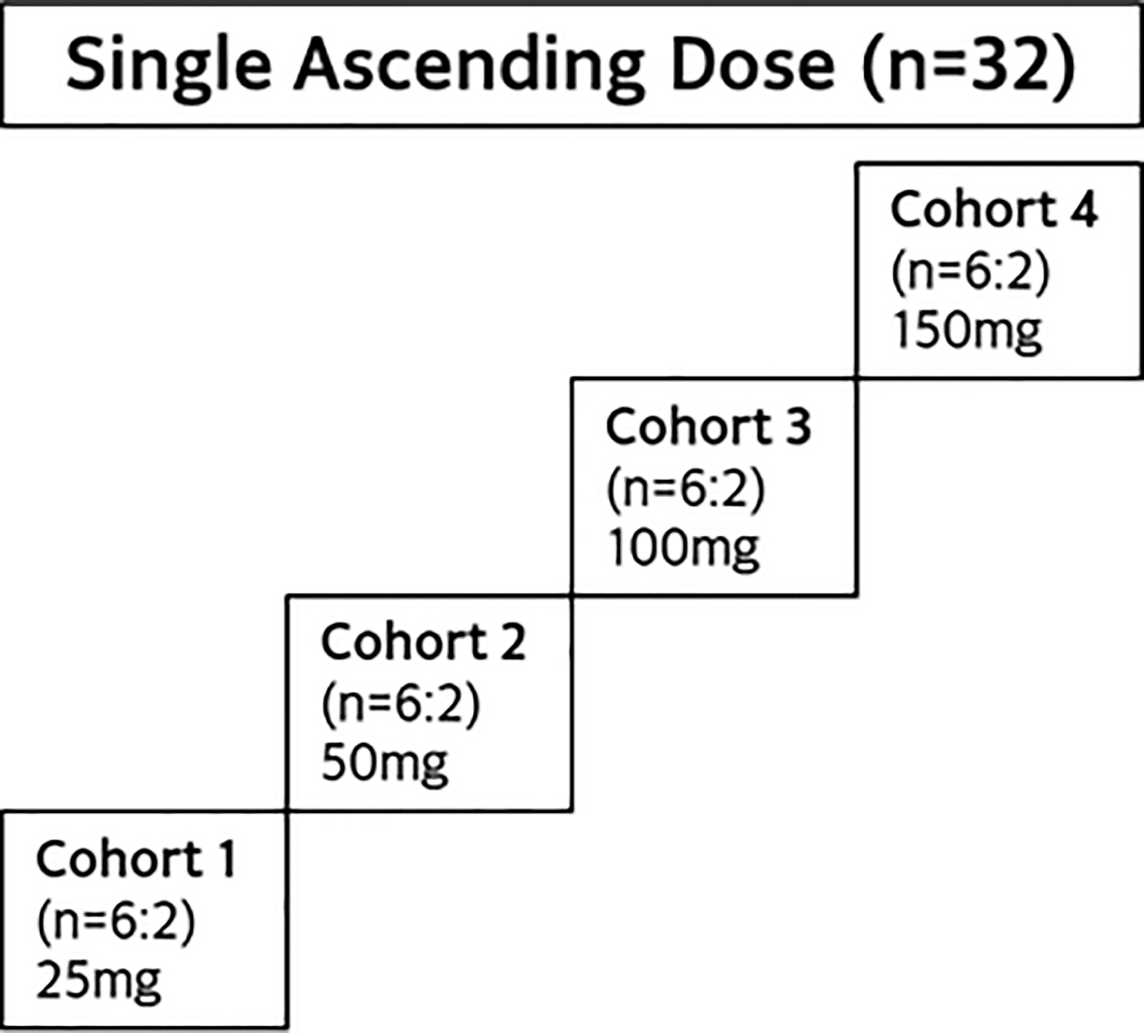
Study design of JACKPOT3.

This was a SAD study in healthy adult Asian participants, with a study design similar to the SAD cohorts of JACKPOT2 study. As shown in **Figure 2**, a total of 4 dose cohorts was planned, with 8 participants in each cohort. A total of 32 healthy participants were randomized to receive a single dose of golidocitinib or matched placebo (6:2).

#### 2.2.2 Participants

Healthy male and female Asian participants were aged 18~45 years old with a body mass index (BMI) between 18 and 28 kg/m^2^. Participants were not included in the study if they had a clinically significant history of hematological, renal, endocrine, pulmonary, gastrointestinal, cardiovascular, hepatic, psychiatric, neurologic, or allergic diseases; history of infections; received a live vaccine within three months before the first dose of golidocitinib; no over-the-counter or prescription medications within 21 days or five half-lives (whichever is longer), prior to receiving golidocitinib. Smoking was prohibited for 30 days prior to receiving golidocitinib and throughout the duration of the study. Alcohol was prohibited from 72 hours before admission for any study-related procedures and during the residential period. Participants were treated under standardized fasted conditions (10 hours before and 4 hours after drug administration).

#### 2.2.3 PK sampling and bioanalytical assays

After single oral administration, serial blood samples were obtained on Day 1 at pre-dose, and at 0.5, 1, 1.5, 2, 3, 4, 6, 8, 12, 24 (Day 2), 48 (Day 3), 72 (Day 4), 120 (Day 6), 168 (Day 8) and 216 (Day 10) hours post-dose for plasma PK analysis. Urine samples were collected during the collection intervals of 0-4, 4-10, 10-24, 24-48, 48-72, 72-96, 96-120, 120-144, and 144-168 hours following a single oral dose of 100 mg for urine PK analysis. Validated LC/MS methods were used for the determination of golidocitinib in human plasma and urine by Frontage Laboratories Inc. (Shanghai). This method was cross validated by Frontage Laboratories Inc. (New Jersey, USA) and met the acceptance criteria.

#### 2.2.4 Safety assessments

The safety monitoring for both JACKPOT2 and JACKPOT3 was similar, included monitoring of adverse events/serious adverse events, clinical safety laboratory tests (clinical chemistry, hematology, coagulation, and urinalysis tests), 12-lead ECG, vital signs (blood pressure, pulse rate, and respiratory rate), and pulmonary function.

### 2.3 Statistical analysis

Assessment of inter-ethnic difference of golidocitinib PK: The primary analyses of AUC_0–t_, AUC_0–inf,_ and C_max_ (log-transformed dose-normalized PK parameters) were performed using an analysis of covariance (ANCOVA) model, with the ethnic group as a fixed effect and dose (log scale) as a covariate. The analysis of variance (ANOVA) model was built for the secondary PK endpoints (log-transformed CL/F, Vz/F, and t_1/2_) with the ethnic group as a fixed effect. The Black/Caucasian and Asian/Caucasian ratios of the geometric mean were calculated by taking the antilogarithm of the difference between group means and corresponding 90% confidence intervals (CIs) in the log scale. Other analyses are descriptive, and no formal statistical comparison is performed for pooled JACKPOT2 and JACKPOT3 data.

## 3 Results

### 3.1 Participants

As the study design of SAD cohorts in JACKPOT2 and JACKPOT3 studies was similar, pooled analysis was performed. The race factor is the focus potentially used for the exposure comparison. A total of 62 participants were included in the analysis (11 Caucasians, 19 Blacks and 32 Asians). The characteristics of Age and BMI are comparable across the race groups with the range 30.5 – 34.6 years old and 24.1 – 25.0 kg/m^2^, respectively, in the participants treated with golidocitinib (**Table 1**). One participant of 150 mg cohort in JACKPOT 2 study was lost to follow-up and did not complete the study in this part.

**Table 1:** Participant demographics and baseline characteristics (SAD cohorts, JACKPOT2 and JACKPOT3).

	Race	n	Age (years)	BMI (kg/m^2^)
Placebo	Caucasian	4	30.3 (8.85)	25.4 (2.51)
	Black	6	31.5 (8.24)	25.1 (1.57)
	Asian	8	27.3 (4.59)	24.4 (2.01)
Golidocitinib	Caucasian	7	34.6 (5.29)	24.1 (2.92)
	Black	13	31.5 (7.92)	25.0 (2.24)
	Asian	24	30.5 (6.37)	24.3 (2.91)

SAD, Single Ascending Dose.

Data are presented as arithmetic mean ± standard deviation.

A total of 12 participants were included in the food effect cohorts and twenty-four participants were included in MAD cohorts. The similar characteristics of demographics were found across the cohorts (**Tables S1, S2**).

### 3.2 Pharmacokinetics

**Figure 3:**
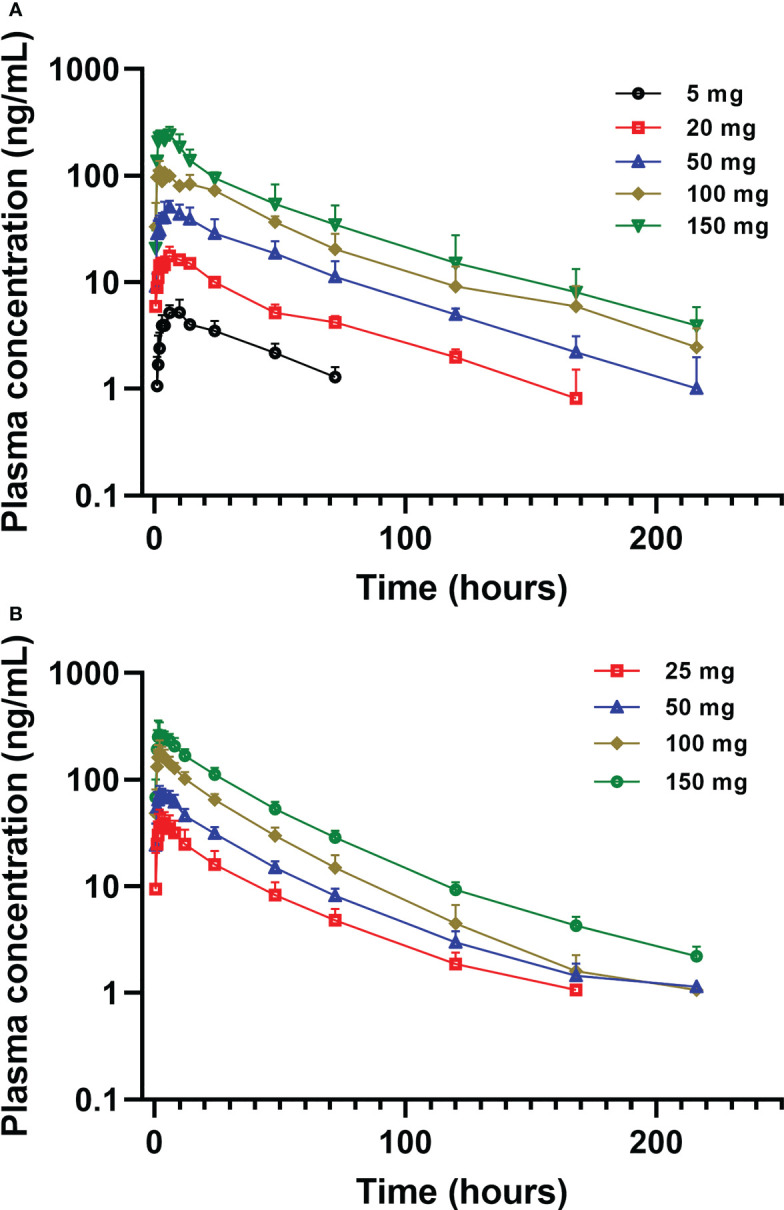
Mean (± SD) plasma concentration of golidocitinib following a single oral dose of golidocitinib: **(A)** subjects were dosed from 5 mg to 150 mg in JACKPOT2 study (n = 4 per dose level); **(B)** subjects were dosed from 25 mg to 150 mg in JACKPOT3 study (n = 6 per dose level). Blood samples were collected up to 216 hours post-dose for each subject.

Mean plasma concentration-time profiles of golidocitinib per treatment and clinical study (linear scale with standard deviation) are shown in **Figure 3**. Following a single oral administration, golidocitinib was absorbed across the dose range with a median t_max_ of 2.50 to 8.00 hours in US participants and 1.75 to 3.00 hours in Chinese participants (**Table 2A**). After reaching C_max_, plasma concentration declined in a multi-exponential manner with a median terminal half-life (t_1/2_) of 42.8 to 51.3 hours and 28.3 to 46.7 hours in these two populations, respectively. The mean clearance from both US and Chinese participants were around 20 L/hr (18.1 – 22.1 L/hr), indicating the low plasma clearance compared to the liver blood flow. Golidocitinib was extensively distributed with mean Vz/F values of 1380 – 1540 L and 825.8 – 1213 L in US and Chinese participants, respectively.

**Table 2A:** Plasma PK parameters of golidocitinib following single or multiple doses.

Following single-dose administration							
Dose (mg)	N	AUC_0-inf_ (hr*ng/mL)	C_max_ (ng/mL)	t_max_ (hour)	t_1/2_ (hour)	CL/F (L/hr)	Vz/F (L)
Phase I study in USA (JACKPOT2)							
5	4	NC	5.14 (22.3)	8.00 (6.00, 10.0)	NC	NC	NC
20	4	923.0 (5.52)	18.6 (14.8)	8.00 (6.00, 10.0)	51.3 (40.8, 52.9)	21.7 (1.18)	1540 (180)
50	4	2340 (29.1)	48.5 (15.8)	6.00 (6.00, 10.0)	42.8 (36.7, 64.0)	22.1 (6.20)	1420 (224)
100	4	5260 (16.4)	133 (20.9)	2.50 (1.00, 4.00)	50.4 (42.1, 55.0)	19.2 (3.09)	1380 (353)
150	4	7260 (39.9)	232 (20.8)	3.00 (2.00, 6.00)	46.6 (23.8, 73.0)	21.9 (8.58)	1400 (624)
Phase I study in China (JACKPOT3)							
25	6	1234 (36.5)	40.8 (33.4)	3.00 (2.00, 6.00)	38.0 (24.4, 39.3)	21.4 (37.6)	1041 (28.2)
50	6	2371 (12.8)	75.0 (12.3)	2.00 (1.00, 6.00)	39.1 (23.7, 50.1)	21.2 (13.1)	1164 (26.0)
100	6	4735 (16.4)	184 (23.6)	2.00 (2.00, 6.00)	28.3 (19.2, 33.7)	21.4 (16.5)	825.8 (18.0)
150	6	8341 (14.9)	280 (21.7)	1.75 (1.50, 6.00)	46.7 (39.5, 53.1)	18.1 (14.2)	1213 (17.8)
On Day 1 and Day 14 of the multiple-dose study (JACKPOT2)							
Dose (mg)	N	Day	AUC_0-24h_ (hr*ng/mL)	C_max_ (ng/mL)	t_max_ (hour)	Rac (C_max_)	Rac (AUC_0-24h_)
25	6	1	536.0 (20.5)	33.8 (22.0)	6.00 (6.00, 6.00)	N/A	N/A
50	6	1	1020 (15.2)	62.1 (14.0)	6.00 (3.00, 6.00)	N/A	N/A
100	6	1	2490 (16.1)	149 (15.0)	4.00 (1.50. 6.00)	N/A	N/A
25	6	14	1530 (16.3)	83.9 (14.2)	6.00 (3.00, 10.0)	2.5 (0.36)	2.9 (0.50)
50	6	14	3190 (11.7)	169 (10.8)	6.00 (6.00, 6.00)	2.8 (0.37)	3.2 (0.34)
100	6	14	7190 (16.4)	375 (13.1)	6.00 (4.00, 6.00)	2.6 (0.49)	2.9 (0.60)

AUC_0-inf_ area under the serum concentration–time curve from time zero to infinity; C_max_ maximum observed serum concentration; t_max_ time to reach maximum observed serum concentration; t_1/2_ apparent terminal half-life; CL/F apparent total clearance; V_z_/F apparent volume of distribution during the terminal phase; AUC_0-24h_ area under the serum concentration–time curve from time zero to 24 hour; Rac accumulation ratio.

Geometric mean (CV%) for AUC_0-inf,_ C_max_ and AUC_0-24h_; Median (min, max) for t_max_ and t_1/2_; Mean (SD) for CL/F, Vz/F, Rac(C_max_) and Rac(AUC_0-24h_).

NC, Not calculable; N/A, Not applicable.

**Table 2B:** Urinary PK parameters of golidocitinib.

Dose level (mg)	N	Ae_0-t_ (mg)	CL_r_ (L/h)	CL_total_ (L/h)	Fe_0-t_ (%)
JACKPOT2 (MAD) following 14-day once daily dose of golidocitinib					
25	6	4.94 (1.23)	3.18 (0.50)	15.2	20.9
50	6	11.7 (1.69)	3.69 (0.68)	15.5	23.9
100	6	19.2 (4.11)	2.71 (0.73)	13.4	20.2
JACKPOT3 (SAD) following single dose of golidocitinib					
100	6	19.5 (6.22)	4.07 (0.99)	15.0	27.2

SAD, Single Ascending Dose; MAD, Multiple Ascending Dose. Data are presented as arithmetic mean or arithmetic mean ± standard deviation.

Ae_0-t_ and Fe_0-t_ are defined as the total excretion amount and percentage of excretion within 0-24 hour collection period on Day 14 from JACKPOT2 study; and within 0-168 hour collection period since Day 1 from JACKPOT3 study.

CLr is defined as renal clearance and estimated from the equation Ae_0-t_/AUC_0-t_.

CL_total_ is derived from apparent plasma clearance (CL/F from **Table 2A**) with F (bioavailability is approximately 0.7, estimated from physiologically based pharmacokinetic (PBPK) model based on the data on file).

**Table 2C:** Statistical analysis of the effect of race on the pharmacokinetic parameters of golidocitinib - single dose in healthy volunteers.

Parameter Race	n	Geometric Mean (CV%)	GLS Mean	Ratio	90% CI
Dose normalized AUC_0-t_ (hr*ng/mL/mg)					
Caucasian	7	42.59 (28.57)	42.52	N/A	N/A
Black	13	46.51 (23.98)	47.72	1.1	(0.93, 1.36)
Asian	24	48.15 (23.34)	47.50	1.1	(0.94, 1.33)
Dose normalized AUC_0-inf_ (hr*ng/mL/mg)					
Caucasian	6^a^	43.62 (23.48)	43.13	N/A	N/A
Black	10^a^	51.54 (22.55)	52.05	1.2	(0.97, 1.46)
Asian	24	49.82 (21.63)	49.76	1.2	(0.98, 1.36)
Dose normalized C_max_ (ng/mL/mg)					
Caucasian	7	1.17 (30.17)	1.16	N/A	N/A
Black	13	1.12 (26.07)	1.19	1.0	(0.85, 1.23)
Asian	24	1.70 (24.14)	1.66	1.4	(1.20, 1.68)
CL/F (L/hr)					
Caucasian	6	22.92 (23.48)	22.92	N/A	N/A
Black	10	19.40 (22.55)	19.40	0.85	(0.70, 1.02)
Asian	24	20.07 (21.59)	20.07	0.88	(0.74, 1.04)
V_z_/F (L)					
Caucasian	6	1351 (19.57)	1351	N/A	N/A
Black	10	1424 (28.01)	1424	1.1	(0.84, 1.33)
Asian	24	1025 (27.61)	1025	0.76	(0.62, 0.93)
t_1/2_ (hour)					
Caucasian	6	40.84 (29.23)	40.84	N/A	N/A
Black	10	50.89 (19.58)	50.89	1.3	(0.99, 1.56)
Asian	24	35.37 (28.21)	35.37	0.87	(0.71, 1.06)

AUC_0-t_, area under the serum concentration–time curve from time zero to time t; AUC_0-inf_, area under the serum concentration–time curve from time zero to infinity; C_max_, maximum observed serum concentration; t_1/2_ apparent terminal half-life; CL/F, apparent total clearance; V_z_/F, apparent volume of distribution during the terminal phase; Kel, elimination rate constant; CI, confidence interval; CV, coefficient of variation; GLS, geometric least square. NA, not applicable.

a Data for participants from 5 mg group were not included. The AUC_0-inf_ could not be calculated because the %AUC_extr_ > 20%.

Geometric mean was calculated as exp (μ), where μ is the mean of the data on a logarithmic scale. Geometric CV% was calculated as [exp(s^2^)-1]^1/2^*100, where s is the standard deviation of the data on a logarithmic scale. For AUC_0-t_, AUC_0-inf_ and C_max_, the analysis of covariance model was built for each log-transformed dose normalized PK parameter separately, with the race as fixed factor and log-transformed dose as covariate. Least square means for country, difference relative to White with 90% CI were estimated and back transformed as the GLS means and ratios in original scale. For CL/F, V_z_/F, and t_1/2_, the analysis of variance model was built for each log-transformed PK parameter separately, with the race as fixed factor. Least square means for country, difference relative to White with 90% CI were estimated and back transformed as the GLS means and ratios in original scale.

The mean (SD) amount of golidocitinib excreted in the urine over the collection period and renal clearance are presented in **Table 2B**. The average amount of golidocitinib excreted in the urine increased in an approximately dose proportional manner from 25 mg to 100 mg. The mean values of corresponding renal clearance were 2.71 to 3.69 L/hr in US participants, and 4.07 L/hr in Chinese participants. The renal excretion fraction (Fe_0-t_) is helpful to compare the renal clearance (CL_r_) with total clearance (CL_total_) and determine the contribution of renal clearance in the drug elimination. It was calculated by the equation Fe_0-t_ = CL_r_/CL_total_. The CL/F values were obtained from the two Phase I studies after the oral administration. Since the pharmacokinetics of golidocitinib after intravenous injection was not studied, the bioavailability value (F) was calculated by physiologically based pharmacokinetic (PBPK) model that incorporated compound specific information and independent prior knowledge on the physiology and biology at the organism. Hence, CL_total_ was calculated by CL/F (from **Table 2A**) * F (0.7, estimated from PBPK model). The Fe_0-t_ values were 20.2% to 23.9% in US participants and 27.2% in Chinese participants, indicating that golidocitinib is moderately eliminated *via* renal excretion as compared with other elimination pathways.

Dose proportionality of AUC and C_max_ was demonstrated following 5 mg to 150 mg (single dose) and 25 mg to 100 mg (QD for 14 days), which is confirmed by power model. As illustrated in **Table S3**, a slope, β, with 90% CI, was 1.04 (0.905 - 1.18) for AUC_0-inf_, and 1.12 (1.04 - 1.19) for C_max_, suggesting that the exposure is approximately dose-proportional for single oral doses ranging from 5 mg to 150 mg. As shown in **Table S4**, a slope, β (90% CI) was 1.11 (0.977-1.24) for AUC_0-24_, and 1.07 (0.939-1.20) for C_max_, also suggesting that the exposure is approximately dose-proportional for multiple doses ranging from 25 mg to 100 mg.

### 3.3 Assessment of inter-ethnic difference of golidocitinib PK

The healthy participants from these two studies included in the ethnicity analysis represented major race groups (Caucasian, Black, and Asian), as shown in **Table 2C**. Overall, primary PK parameters show comparable or slightly higher AUC values observed in Asian or Black participants compared to Caucasian participants for AUC_0-inf_ ratio of 1.2. In contrast, higher C_max_ was observed in Asian healthy adults compared to Caucasians with a C_max_ ratio of 1.4.

### 3.4 Food effect

Following single-dose administration (50 mg) of golidocitinib under fasted and fed conditions, plasma concentrations were quantifiable up to 216 hours post-dose. The plasma concentration of golidocitinib was comparable between the fed and fasted conditions. The median t_max_ was 6.00 hours under both fasted and fed conditions, which indicates the high-fat/high-calorie diet doesn’t increase the drug absorption rate. The geometric mean of C_max_ under fasted and fed conditions were 68.8 and 73.7 ng/mL, respectively. The geometric mean of AUC_0-t_ in the fasted and fed condition were 2770 hr*ng/mL and 3230 hr*ng/mL, respectively. The results of C_max_ and AUC_0-t_ demonstrated that the high-fat/high-calorie diet has minimal effect on the exposure of golidocitinib in healthy participants.

The mixed-effects model was used to evaluate the food effect in this study. The result is presented in **Table 3**. The percent ratio of geometric least square means and 90% CI of C_max_, AUC_0-t,_ and AUC_0-∞_ were 108.9 (97.81, 121.2), 115.0 (109.3, 121.0) and 116.5 (111.2, 122.0) under fed versus fasted condition, which showed the high-fat/high-calorie diet had minimal effect on the exposure of golidocitinib in healthy participants.

**Table 3:** Statistical analysis of the effect of food on the PK parameters of golidocitinib.

Golidocitinib	Fasted (Reference) (N = 12)	High-fat meal (Test) (N = 11)	Geometric Mean Ratio (90% CI) (Test vs Reference)
t_max,_ hour Median (range)	6.00 (3.00, 14.0)	6.00 (4.00, 14.0)	N/A
C_max_, ng/mL Geometric mean (%CV)	68.8 (25.6)	73.7 (18.4)	108.9 (97.81-121.2)
AUC_0-t_, hr*ng/mL Geometric mean (%CV)	2770 (16.9)	3230 (19.2)	115.0 (109.3-121.0)
AUC_0-inf_, hr*ng/mL Geometric mean (%CV)	2960 (16.5)	3400 (18.7)	116.5 (111.2-122.0)

AUC_0-t_, area under the serum concentration–time curve from time zero to time t; AUC_0-inf_, area under the serum concentration–time curve from time zero to infinity; C_max_, maximum observed serum concentration; t_max_, time to reach maximum observed serum concentration; CI, confidence interval; CV, coefficient of variation. NA, not applicable.

### 3.5 Safety

A total of 98 healthy participants were enrolled in these two clinical studies. Golidocitinib was well-tolerated in healthy participants, with doses ranging from 5 mg to 150 mg in single-dose cohorts and 25 mg to 100 mg in multiple-dose cohorts. Most TEAEs observed in healthy volunteer studies were mild or moderate. According to the investigators’ assessment, no CTCAE grade 3 or higher drug-related TEAE was reported. No serious adverse events were reported. In the food effect part of JACKPOT2 study, one subject was discontinued due to an AE of trigeminy after receiving 50 mg golidocitinib in the fasted state. The AE was mild and resolved within 2 hours without treatment. All TEAEs reported in these two studies are summarized in **Supplementary Tables S5-S8**.

## 4 Discussion

These two studies investigated the PK, safety, and tolerability profile of golidocitinib in healthy adult subjects. The initial starting dose of 5-mg in the First-Time-in-Human study (JACKPOT2) was based on the nonclinical toxicology; Furthermore, PK and pharmacology were also taken into considerations. The initial dose administrated to a subject would be equal to or lower than either the dose calculated following the draft FDA guideline (Guidance for Industry; Estimating the Maximum Safe Starting Dose in Initial Clinical Trials for Therapeutics in Adult Healthy Volunteers, July 2005), or a dose that is predicted to elicit a minimal pharmacodynamic (PD) effect. No Observed Adverse Effect Level (NOAEL) of 6 mg/kg is determined in the most sensitive species (rat) in one-month toxicology study. The NOAEL toxicology dose is then converted to the Human Equivalent Dose (HED) of 0.972 mg/kg based on body surface area (BSA) conversion. A safety margin of 10-fold was applied to estimate the recommended safe starting dose of 5 mg. Moreover, it is not anticipated to have pharmacological effect based on *in vitro* pharmacology data and predicted PK. With the reference of safety data from JACKPOT2 SAD cohorts, higher starting dose of 25 mg was selected in the next JACKPOT3 study.

Dose proportionality was demonstrated for C_max_ and AUC across the range of a single dose of up to 150 mg and once-daily dosing of up to 100 mg. As expected of long half-life, steady-state plasma concentrations of golidocitinib were achieved by day 14 with once-daily dosing. A moderate accumulation of golidocitinib was observed after multiple dosing; C_max_ and AUC_0-24hr_ on day 14 increased by 2-3 folds compared to day 1. The estimated glomerular filtration rate (GFR) is 37.3%*7.5 hr/L (free fraction in plasma*GFR) = 2.80 hr/L, comparable to or slightly lower than the renal clearance. This indicates that active transport contributes to a minor extent to the overall elimination of golidocitinib. In the first part of JACKPOT2, a single dose of 50 mg was selected because the limited data available when this study was planned indicated that this therapeutic dose had acceptable safety and tolerability. The 90% CI for the geometric least square mean (GLSM) ratio (fed/fasted) of C_max_, AUC_0-t_, and AUC_0-inf_ was totally contained within the range of 0.80 to 1.25, suggesting that high-fat/high-calorie food has minimal effect on the PK of golidocitinib. Compared to approved JAK inhibitors (Tofacitinib, Filgotinib, Upadacitinib) (13), golidocitinib demonstrated a more selective profile of the JAK family and a much longer half-life. It is critical when sustained modulation of JAK/STAT3 pathways is required for efficacy outcomes in refractory and relapsed PTCL indication. A thorough characterization of PK profiles is necessary for dose exploration and optimization for subsequent studies performed in the patients to attain and maintain appropriate plasma exposure.

The inter-ethnic difference in primary PK parameters was evaluated, which suggested slightly higher C_max_ but comparable AUC was observed in Asian subjects as compared to Caucasian and/or Black subjects, while it is not expected that variability affects the efficacy and safety profiles to clinically meaningful extents. The following properties of golidocitinib make it less likely to be sensitive to ethnic factors with the reference of ICH E5 guidance: (1) PK is dose-proportional from 5 mg to 150 mg following a single oral dose; (2) Multiple pathways are involved in clearance of golidocitinib, which mitigate the potential interaction as a victim. Available data showed a primary hepatic clearance (up to 70-80%) with moderate involvement of renal excretion (approximately 20-30%). Moreover, two separate enzymatic systems implicated in the metabolism of golidocitinib were CYP3A4/5 and flavin-containing monooxygenase enzyme (FMO); (3) High oral bioavailability (>80%) can be concluded in the view of the metabolic profile of golidocitinib in human from a radiolabeled mass balance study in 8 healthy participants (JACKPOT5, NCT04225208). Additionally, golidocitinib has high permeability and high solubility (the clinical dose remains soluble in the intestinal fluid of 250 mL). Thus, efflux of golidocitinib *via* gut transporters is unlikely to limit oral absorption; (4) Only ~ 63% of golidocitinib is bound to human plasma of Caucasian and Chinese *in vitro*. Thus fluctuations of alpha1 acid glycoprotein (AAG) in plasma between ethnic groups are unlikely to affect free drug exposure; (5) Diet unlikely affect the PK of drugs to a clinically meaningful extent in the view of clinical investigation with a high-fat meal; (6) genetic polymorphism of metabolizing enzymes less likely affect the PK of the drug because of multiple pathways involved in metabolism and excretion, neither polymorphism of transporters impacts the PK of golidocitinib because of high passive permeability and high solubility.

Following single oral administration, golidocitinib is absorbed more slowly with larger t_max_ at lower doses than at higher doses. *In vitro* experiments showed that golidocitinib is a P-glycoprotein (P-gp) substrate, but active transport can be saturable at high concentrations. Additionally, golidocitinib has high solubility and high permeability *in vitro*. Thus, delayed t_max_ at the low dose levels may be caused by active efflux and metabolism *via* gut P-gp and CYP3A4; however, high permeability and high solubility of golidocitinib minimizes the impact but ends with lower C_max_ and comparable AUC at the same dose levels between JACKPOT2 and JACKPOT3 studies. Drugs undergoing active transport, efflux of P-gp, and gut metabolism *via* CYP3A4 are most likely to show the inter-ethnic difference in drug absorption (14–16). These observations may be associated with polymorphism and/or expression differences between major ethnic groups. However, no clear studies show variations in drug absorption between Asians and Caucasians. The interplay between CYP3A4 and P-gp and high variable expression among individuals make it difficult to elucidate the ethnic difference in oral absorption of golidocitinib. It was speculated that C_max_ difference would be minimized following multiple dosing due to accumulation. Therefore, the absorption phase of the single dose will contribute less to C_max_ at steady state.

Single doses of golidocitinib up to 150 mg and 14-day dosing up to 100 mg once daily were well-tolerated in Asian and non-Asian healthy adult participants. In these two studies, TEAEs were mainly mild or moderate in severity and were reversible without any clinical sequelae. There were no golidocitinib-related Grade 3 or higher TEAEs or SAEs. Moreover, vital signs, ECG, and laboratory parameters showed no consistent clinically relevant trends or changes. Overall, no vital risk of golidocitinib was identified in both studies. The safety of golidocitinib will be further evaluated in ongoing studies in patients with PTCL (ClinicalTrials.gov Identifier: NCT04105010).

These studies had several limitations. Firstly, the inter-ethnic comparison is based on PK data of golidocitinib from the SAD cohorts among these ethnic groups. It is currently unknown whether multiple-dose PK behaves similarly or not between these races. Secondly, other limitations include the short duration (14-day) in the MAD cohorts relative to the long half-life and the inclusion of healthy subjects instead of cancer patients. The study population was young and not representative of the age demographic of the target population for which golidocitinib is intended. Long-term safety was investigated in the phase I/II study performed in patients with NSCLC (ClinicalTrials.gov Identifier: NCT03450330) and r/r PTCL, in which PK, PD, and safety assessments were assessed in patients with different ethnic backgrounds.

The major strength of JACKPOT2 and JACKPOT3 is that they provided a thorough demonstration of golidocitinib PK behavior. It was demonstrated that no racial differences and food effects were observed in these two studies. Further investigations of PK in patients, as well as population PK modeling, are required to corroborate the results here.

## 5 Conclusion

No inter-ethnic difference in PK was observed among healthy Caucasian, Black, and Asian subjects. Secondly, high-fat/high-calorie food had minimal effect on the PK of golidocitinib. Thus, data accumulated so far did not suggest different dosing regimens in the intended patient population for multinational clinical studies. Importantly, golidocitinib was well tolerated in healthy participants, which warrants further clinical development.

## Data availability statement

The original contributions presented in the study are included in the article/**Supplementary Material**. Further inquiries can be directed to the corresponding author.

## Ethics statement

JACKPOT2 study were conducted in Frontage Clinical Research Center in Secaucus, New Jersey, USA. JACKPOT3 study was conducted in Peking Union Medical College Hospital in Beijing, China. Both the studies were performed in compliance with the ethical principles originating in, or derived from, the Declaration of Helsinki, as well as in compliance with all International Conference on Harmonization Good Clinical Practice guidelines. In addition, all local regulatory requirements were followed, and JACKPOT2 study was registered on ClinicalTrials.gov (NCT03728023) and JACKPOT3 on cde.org.cn (CTR20191011). JACKPOT2 study was approved by IntegReview IRB and ethics committee. JACKPOT3 was approved by the Institutional Review Board and ethics committee in Peking Union Medical College Hospital. All participants provided written, informed consent before joining the studies.

## Author contributions

RC, KC, and ZY designed and organized the clinical trial. KC analyzed the data and wrote the manuscript. KC, XG, YZ, ZL, XD and DL were involved in the discussion of results. RC, KC, ZY, YZ, ZL, XD, and PH reviewed and edited the manuscript. All the authors have read and approved the final manuscript. All authors contributed to the article and approved the submitted version.
