# Supplementary material for: Pharmacokinetic characteristics of golidocitinib, a highly selective JAK1 inhibitor, in healthy adult participants
Source: Front Immunol. 2023 Apr 3;14:1127935. doi: 10.3389/fimmu.2023.1127935 (PMC10108266; doi:10.3389/fimmu.2023.1127935)
Supplement: Supplementary file 1 [file DataSheet_1.docx]

Supplementary Material

# Supplementary Tables

**Table S1. Participant demographics (Food effect cohorts, JACKPOT2)**

|  | n | Age (years) | BMI (kg/m^2^) |
| --- | --- | --- | --- |
| Golidocitinib (Fasted) | 12 | 33.8 (6.24) | 26.4 (2.88) |
| Golidocitinib (Fed) | 11 | 33.9 (6.52) | 26.1 (2.88) |

Data are presented as arithmetic mean ± standard deviation.

**Table S2. Participant demographics (MAD cohorts, JACKPOT2)**

|  | n | Age (years) | BMI (kg/m^2^) |
| --- | --- | --- | --- |
| Placebo | 6 | 35.8 (4.12) | 26.2 (3.06) |
| Golidocitinib (25 mg) | 6 | 34.0 (6.78) | 25.5 (2.24) |
| Golidocitinib (50 mg) | 6 | 32.5 (4.68) | 27.2 (1.97) |
| Golidocitinib (100 mg) | 6 | 32.0 (6.26) | 25.0 (2.20) |

MAD: Multiple Ascending Dose;

Data are presented as arithmetic mean ± standard deviation.

| Table S3. Golidocitinib dose proportionality assessment (SAD cohorts) | | | |
| --- | --- | --- | --- |
| Parameter (unit) | β (Slope) | Standard Error | 90% CI |
| AUC_0-inf_ (hr*ng/mL) | 1.04 | 0.0782 | 0.905-1.18 |
| AUC_0-t_ (hr*ng/mL) | 1.03 | 0.0461 | 0.953-1.11 |
| C_max_ (ng/mL) | 1.12 | 0.0414 | 1.04-1.19 |

| Table S4. Golidocitinib dose proportionality assessment (MAD cohorts) | | | |
| --- | --- | --- | --- |
| Parameter (unit) | β (Slope) | Standard Error | 90% CI |
| AUC_0-24_ (hr*ng/mL) | 1.11 | 0.0745 | 0.977-1.24 |
| C_max_ (ng/mL) | 1.07 | 0.0749 | 0.939-1.20 |

| Table S5. TEAEs by SoC, PT and Treatment – SAD (JACKPOT2) | | | | | | |
| --- | --- | --- | --- | --- | --- | --- |
| System Organ Class (SoC)  Preferred Term (PT), n (%) | Placebo | Golidocitinib | | | | |
|  |  | 5 mg | 20 mg | 50 mg | 100 mg | 150 mg |
|  | n = 10 | n = 4 | n = 4 | n = 4 | n = 4 | n = 4 |
| Subjects with at least one TEAE | 3 (30.0%) | 2 (50.0%) | 2 (50.0%) | 1 (25.0%) | 1 (25.0%) | 0 |
| Blood and Lymphatic System Disorders | 1 (10.0%) | 2 (50.0%) | 2 (50.0%) | 0 | 1 (25.0%) | 0 |
| Anemia | 0 | 1 (25.0%) | 0 | 0 | 0 | 0 |
| Eosinophilia | 1 (10.0%) | 2 (50.0%) | 0 | 0 | 0 | 0 |
| Neutropenia | 1 (10.0%) | 0 | 1 (25.0%) | 0 | 0 | 0 |
| Thrombocytosis | 0 | 0 | 1 (25.0%) | 0 | 1 (25.0%) | 0 |
| General Disorders and Administration Site Conditions | 0 | 0 | 0 | 0 | 1 (25.0%) | 0 |
| Chest Discomfort | 0 | 0 | 0 | 0 | 1 (25.0%) | 0 |
| Investigations | 1 (10.0%) | 0 | 0 | 1 (25.0%) | 0 | 0 |
| ALT Increased | 1 (10.0%) | 0 | 0 | 0 | 0 | 0 |
| AST Increased | 1 (10.0%) | 0 | 0 | 0 | 0 | 0 |
| N-Terminal Pro‑BNP | 0 | 0 | 0 | 1 (25.0%) | 0 | 0 |
| Respiratory, Thoracic and Mediastinal Disorders | 1 (10.0%) | 2 (50.0%) | 0 | 0 | 0 | 0 |
| Dyspnea | 1 (10.0%) | 0 | 0 | 0 | 0 | 0 |
| Nasal Congestion | 0 | 1 (25.0%) | 0 | 0 | 0 | 0 |
| Oropharyngeal Pain | 0 | 1 (25.0%) | 0 | 0 | 0 | 0 |

| Table S6. TEAEs by SoC, PT and food effect (JACKPOT2) | | |
| --- | --- | --- |
| System Organ Class (SoC) Preferred Term (PT), n (%) | Golidocitinib 50 mg (Fasted) | Golidocitinib 50 mg (Fed) |
|  | n = 12 | n = 11 |
| Subjects with at least one TEAE | 3 (25.0%) | 7 (63.6%) |
| Blood and Lymphatic System Disorders | 0 | 1 (9.1%) |
| Anemia | 0 | 1 (9.1%) |
| Cardiac Disorders | 1 (8.3%) | 0 |
| Extrasystoles | 1 (8.3%) | 0 |
| Investigations | 2 (16.7%) | 4 (36.4%) |
| Alanine Aminotransferase Increased | 1 (8.3%) | 3 (27.3%) |
| Aspartate Aminotransferase Increased | 0 | 2 (18.2%) |
| Blood Lactate Dehydrogenase Increased | 0 | 1 (9.1%) |
| Lipase Increased | 1 (8.3%) | 1 (9.1%) |
| Nervous System Disorders | 0 | 1 (9.1%) |
| Headache | 0 | 1 (9.1%) |
| Respiratory, Thoracic and Mediastinal Disorders | 0 | 2 (18.2%) |
| Cough | 0 | 1 (9.1%) |
| Rhinorrhea | 0 | 2 (18.2%) |

MedDRA version 22.0.

Aberration: n (%), number and percent of subjects in the specified group. n, number of subjects in the specified study population.

| Table S7. TEAEs by SoC, PT and Treatment – MAD (JACKPOT2) | | | | |
| --- | --- | --- | --- | --- |
| System Organ Class (SoC) Preferred Term (PT), n (%) | Placebo | Golidocitinib (Fasted) | | |
|  |  | 25 mg | 50 mg | 100 mg |
|  | n = 6 | n = 6 | n = 6 | n = 6 |
| Subjects with at least one TEAE | 2 (33.3%) | 2 (33.3%) | 1 (16.7%) | 3 (50.0%) |
| Blood and Lymphatic Disorders | 0 | 1 (16.7%) | 0 | 3 (50.0%) |
| Leukopenia | 0 | 0 | 0 | 2 (33.3%) |
| Neutropenia | 0 | 1 (16.7%) | 0 | 3 (50.0%) |
| Reticulocytopenia | 0 | 0 | 0 | 1 (16.7%) |
| Gastrointestinal Disorders | 0 | 1 (16.7%) | 1 (16.7%) | 0 |
| Abdominal pain upper | 0 | 0 | 1 (16.7%) | 0 |
| Constipation | 0 | 1 (16.7%) | 1 (16.7%) | 0 |
| Nausea | 0 | 0 | 1 (16.7%) | 0 |
| Injury, Poisoning and Procedural Complications | 1 (16.7%) | 0 | 0 | 0 |
| Skin Laceration | 1 (16.7%) | 0 | 0 | 0 |
| Nervous System Disorders | 0 | 0 | 1 (16.7%) | 1 (16.7%) |
| Headache | 0 | 0 | 1 (16.7%) | 1 (16.7%) |
| Respiratory, Thoracic and Mediastinal Disorders | 1 (16.7%) | 0 | 0 | 0 |
| Dry throat | 1 (16.7%) | 0 | 0 | 0 |

| **Table S8.** TEAEs by SoC, PT and Treatment – MAD (JACKPOT3) | | | | | |
| --- | --- | --- | --- | --- | --- |
| System Organ Class (SoC)  Preferred Term (PT), n (%) | Placebo | Golidocitinib (Fasted) | | | |
|  |  | 25 mg | 50 mg | 100 mg | 150 mg |
|  | n = 8 | n = 6 | n = 6 | n = 6 | n = 6 |
| Subjects with at least one TEAE | 3 (37.5%) | 4 (66.7%) | 3 (50.0%) | 4 (66.7%) | 6 (100%) |
| Investigations | 1 (12.5%) | 2 (33.3%) | 3 (50.0%) | 2 (33.3%) | 4 (66.7%) |
| C-reactive protein increased | 0 | 1 (16.7%) | 0 | 1 (16.7%) | 1 (16.7%) |
| Blood bilirubin increased | 0 | 0 | 1 (16.7%) | 0 | 2 (33.3%) |
| Neutrophil count decreased | 1 (12.5%) | 1 (16.7%) | 0 | 0 | 2 (33.3%) |
| ALT increased | 0 | 0 | 1 (16.7%) | 1 (16.7%) | 0 |
| LDL increased | 0 | 0 | 1 (16.7%) | 0 | 1 (16.7%) |
| AST increased | 0 | 0 | 2 (33.3%) | 0 | 0 |
| Glucose urine present | 0 | 0 | 0 | 0 | 1 (16.7%) |
| ECG QT prolonged | 0 | 0 | 0 | 0 | 1 (16.7%) |
| Blood cholesterol increased | 0 | 0 | 0 | 0 | 1 (16.7%) |
| Hemoglobin decreased | 1 (12.5%) | 0 | 0 | 0 | 1 (16.7%) |
| LDH increased | 0 | 0 | 1 (16.7%) | 0 | 0 |
| Metabolism and nutrition disorders | 2 (25.0%) | 0 | 1 (16.7%) | 2 (33.3%) | 2 (33.3%) |
| Hypertriglyceridemia | 2 (25.0%) | 0 | 1 (16.7%) | 2 (33.3%) | 2 (33.3%) |
| Respiratory, thoracic and mediastinal disorders | 0 | 1 (16.7%) | 0 | 1 (16.7%) | 1 (16.7%) |
| Rhinorrhea | 0 | 0 | 0 | 0 | 1 (16.7%) |
| Epistaxis | 0 | 0 | 0 | 1 (16.7%) | 0 |
| Productive cough | 0 | 1 (16.7%) | 0 | 0 | 0 |
| Gastrointestinal disorders | 0 | 2 (33.3%) | 0 | 0 | 1 (16.7%) |
| Diarrhea | 0 | 1 (16.7%) | 0 | 0 | 1 (16.7%) |
| Abdominal discomfort | 0 | 1 (16.7%) | 0 | 0 | 0 |
| Injury, poisoning and procedural complications | 0 | 1 (16.7%) | 0 | 0 | 1 (16.7%) |
| Wound | 0 | 0 | 0 | 0 | 1 (16.7%) |
| Limb injury | 0 | 1 (16.7%) | 0 | 0 | 0 |
| Musculoskeletal and connective tissue disorders | 0 | 0 | 0 | 1 (16.7%) | 1 (16.7%) |
| Musculoskeletal pain | 0 | 0 | 0 | 0 | 1 (16.7%) |
| Musculoskeletal stiffness | 0 | 0 | 0 | 1 (16.7%) | 0 |
| Blood and lymphatic system disorders | 0 | 1 (16.7%) | 0 | 0 | 0 |
| Leukocytosis | 0 | 1 (16.7%) | 0 | 0 | 0 |

MedDRA version 22.0.

Aberration: n (%), number and percent of subjects in the specified group. n, number of subjects in the specified study population. ALT, alanine aminotransferase. LDL, low density lipoprotein. AST, aspartate aminotransferase. ECG, electrocardiogram. LDH, blood lactate dehydrogenase.
